# Mapping the patient journey of adult patients with Spinal Muscular Atrophy in Greece: key challenges and priorities for action

**DOI:** 10.1186/s13023-025-04165-6

**Published:** 2026-01-21

**Authors:** Christina Golna, Chara Tzavara, Christiana Vasileiadi, Pavlos Golnas, Kornilia Binou, Antigone Karras, Kyriakos Souliotis

**Affiliations:** 1 Health Policy Institute, 8 Agisilaou Str., Maroussi, 15123 Greece; 2 MDA Hellas, 6 Elpidos Str., Athens, 10434 Greece; 3 Department of Social and Educational Policy, University of Peloponnese, Damaskinou and Kolokotroni Str., Corinth, 20100 Greece

**Keywords:** Patient journey, Spinal muscular atrophy, Quality of life, Health policy

## Abstract

**Background:**

Spinal Muscular Atrophy (SMA) carries a heavy burden for patients and caregivers, which, in Greece, is exacerbated by resource constraints and limited care integration. To-date, there were only anecdotal descriptions of - mostly pediatric - SMA patient journey challenges. This survey amongst a population of adult patients with SMA (May – September 2023) reports on their journey’s challenges and supports advocacy to improve their experience and outcomes.

**Methods:**

A novel questionnaire was developed, which recorded basic sociodemographic characteristics, disease history, disease-related financial burden, and mapped the full patient journey per disease phase. In addition, participants self-assessed their functional autonomy and quality of life (QoL). All adults diagnosed and living with SMA were eligible to participate. Participants received an invitation by MDA to complete the questionnaire online. Multiple linear regression analysis was used to find factors associated with QoL and functional autonomy.

**Results:**

Sixty-seven adult SMA patients with a mean age of 35.9 years participated in the survey. Results confirm their complicated journey and the related financial and QoL burden the condition results in. Participants spent an average of over EUR 300 a month for their condition, primarily on physiotherapy sessions, therapy aids and food supplements, and this impacted on their QoL. Higher education was positively correlated with QoL, underlying the importance of early SMA patient integration in school life.

**Discussion:**

This survey reports patient journeys of adult SMA patients and as such adds to the more extensive literature on parents’ or caregivers’ experience with pediatric SMA. There is a need to showcase adult SMA patients’ experience with care and the impact the condition has on their financial outlook and QoL. All data are self-reported. Some refer to events over a decade ago and may, thus, be affected by recall bias.

**Conclusions:**

As SMA management evolves with the wider availability of novel treatments and patients live well into adulthood, so must healthcare systems reconsider patient pathways to ensure patients optimize use of available resources in the most efficient manner.

## Introduction

Spinal muscular atrophy (SMA) is a rare, autosomal recessive neuromuscular disorder occurring in approximately 1 in 10,000 live births. Characterized by degeneration of motor neurons, SMA leads to progressive muscle weakness and atrophy [1]. SMA is caused by mutations in the survival of motor neuron 1 (SMN1) gene on chromosome 5q. Classically, SMA is subdivided into different types according to maximum motor function achieved, with type I to III being the most frequent forms with pediatric onset. In type I the onset is before 6 months of age and the ability to sit independently is not achieved. In type II the onset is between 6 and 18 months; type II children achieve the ability to sit but not to walk independently. Type III patients achieve the ability to walk independently, and the onset is after 18 months [2].

SMA results in a heavy burden for patients, family and caregivers and the health care system. Due to its rare nature and its management challenges, which have been traditionally coupled with limited knowledge and understanding of the condition by physicians across the globe, it became evident that there was a need to establish a wide communication for common standards of care. These, first introduced in 2007 and updated in 2018, detail the minimal care and support that anyone with SMA should receive, irrespective of where they live and the level of resources the country may have to invest in managing SMA [3–5]. They were further supplemented by extensive literature that focused on aspects of quality of care of patients and caregivers [6–12] as well as the need for collaboration between different health care providers to improve management gaps and challenges associated with SMA.

These standards of care became outdated following the authorization of several novel pharmaceutical treatments including gene therapies [8] that impact not only the life expectancy of patients with SMA but also their morbidity and burden from their disease. As patients live longer and transition to adult life (something that was unimaginable some decades back), the challenges related to their care also evolve and require updates, particularly as cross-sectoral collaboration and care integration become even more prominent [5, 13].

In Greece, a country traditionally plagued by lack of integration of health care services [14], geographical disparities in access to care [15], high out of pocket payments [16, 17] and concentration of expertise in very few, select centers, the challenges faced by patients with SMA and their caregivers as they journey through their rare condition, appear even greater than those reported in the literature. It is noted that according to Muscular Dystrophy Association (MDA) Hellas estimates, approximately 150 people live with SMA in Greece (1,5 per 100,000 population [18]), whereas there are an additional 1–6 live births (8 per 100,000 live births [18]), with SMA each year. Nonetheless, SMA patient (and carer) experience with the healthcare system has to-date only being reported anecdotally, in reference to individual patient journeys, primarily in the press. Though extremely important, individual patient journeys do not allow for the generalization of challenges faced by SMA patients, in order for these to be translated into advocacy for specific interventions towards the State.

In addition, Greece has been recovering from a 15-year long struggle with an economic crisis, which was followed by the COVID-19 healthcare emergency. Health care spending and policy planning have been driven by a need to abide to fiscal targets and horizontal cuts across the sector [19] have challenged the sustainability and viability of the system. In light of systemic challenges, SMA management has been limited to ensuring the availability of innovative treatments, including gene therapies, in Greece. It has, to-date, not addressed in a comprehensive manner the optimal patient pathway to identify gaps in current patient experience that impact both system efficiency and patient and family resilience.

To address this gap, MDA Hellas together with the Health Policy Institute, organized a patient journey mapping survey targeted to both adult patients living with SMA and caregivers of pediatric SMA patients. The aim of the survey, which ran from May to September 2023, was to map the journey of a patient with SMA, from first symptom to current day, to define disease burden in terms of both quality of life (QoL) and functional independence as well as financial burden of both patient and family and identify those areas in the journey that require advocacy to measurably improve patient experience and outcomes. This manuscript presents and discusses results from the adult patients’ survey.

## Materials and methods

Participants, all members and friends of MDA Hellas, received an invitation by MDA to complete the survey questionnaire online.

### Survey tool

A novel tool (questionnaire) was developed and customized to the survey audience, i.e., one for adult patients and one for parents and/or unpaid caregivers of pediatric patients. With regards to the adult patients’ survey, the first part of the questionnaire recorded basic sociodemographic characteristics and disease history. The second part of the questionnaire mapped the full patient journey across a continuum, per disease phase, i.e., (a) before and during diagnosis, (b) during transition to adult care and (c) to disease management to-date. In addition, participants were requested to assess their status regarding: (a) functional autonomy, using the validated Barthel Index scale [20], a 10-item scale that measures independence in personal care and mobility tasks, and (b) QoL, using EQ-5D-5 L, a descriptive index comprising five dimensions, i.e., mobility, self-care, usual activities, pain/discomfort, and anxiety/depression, each with five levels of severity, & EQ -VAS, a visual analog scale, where respondents rate their overall health on a scale of 0 to 100 [21]. Finally, the questionnaire recorded disease-related personal or family financial burden.

### Data management processes

Questionnaires were prepared by the Health Policy Institute and reviewed and approved by MDA Hellas. Questionnaires were fully anonymized, and responses could not be traced back to participants by those performing the analysis. Great care has been exercised to promote use of ranges to responses to all questions for which this was deemed appropriate. The Health Policy Institute, which was scientifically responsible for the analysis of responses, never collected, acquired access to, stored or processed non-anonymized respondent data.

### Pilot survey

Questionnaires were sent by MDA Hellas to 4 adult patients to pilot their completion and test content validity and completeness. The pilot identified 2 technical improvements to be incorporated in the online questionnaire, developed on Jotform, which were duly performed prior to finalizing the survey tool. As proposed improvements did not refer to the content or the structure of the survey tool, pilot survey responses were accounted for in total survey sample.

### Statistical analysis

Quantitative variables were expressed as mean values (Standard Deviation) and as median (interquantile range), while categorical variables were expressed as absolute and relative frequencies. Pearson correlations coefficients (r) were used to explore the association of two continuous variables. Multiple linear regression analysis was used with dependent the Barthel index and the EQ-5D-5 L index value, in a stepwise method (p for entry 0.05, p for removal 0.10). Adjusted regression coefficients (β) with standard errors (SE) were computed from the results of the linear regression analyses, as well as F-values and coefficients of determination (R^2^) derived from linear regression analyses for model evaluation. All reported p values are two-tailed. Statistical significance was set at *p* < 0.05 and analyses were conducted using SPSS statistical software (version 26.0).

## Results

Sample consisted of 67 participants (58.2% men) with mean age 35.9 years (SD = 11.1 years). Their characteristics are presented in Table 1. Most participants were university alumni (40.3%) and unmarried (85.1%). 32.8% were unemployed. More than half of the sample (53.7%) had an annual family income of EUR 10,000–30,000 and only 11.9% had private health insurance. Moreover, 34.3% of the sample were less than 10 km or 1–50 km away from the hospital, at which they were regularly attended.

**Table 1:** Sample characteristics (*n* = 67)

	*n*	%
Age, mean (SD)	35.9 (11.1)	
Gender		
Men	39	58.2
Women	28	41.8
Education level		
Primary school	2	3.0
Secondary School	4	6.0
High School	18	26.9
University	27	40.3
MSc /PhD	16	23.9
Employment status		
Employee in public sector	9	13.4
Employee in private sector (full time)	5	7.5
Employee in private sector (part time)	2	3.0
Freelancer	6	9.0
Stay at home	1	1.5
Unemployed	22	32.8
Retired	10	14.9
Other‎	12	17.9
Family status		
Unmarried	57	85.1
Married	10	14.9
Annual family income		
Less than EUR 10,000	22	32.8
EUR 10,000–30,000	36	53.7
EUR 30,000–60,000	8	11.9
Greater than EUR 60,000	1	1.5
Do you have private health insurance?	8	11.9
What is the distance in kilometers between your permanent residence and the hospital, in which you are attended?		
Shorter than 10 km	23	34.3
10–50 km	23	34.3
50–100 km	5	7.5
Greater than 100 km	16	23.9

Participants’ disease characteristics, their caregiver’s characteristics, information from their first symptom to diagnosis and information on their current care/treatment are presented in Table 2. Mean disease duration was 28.9 years (SD = 11.3 years) and 59.7% had SMA type II. The majority of the sample (74.6%) were currently under treatment, primarily for managing their symptoms (49.3%) or with a disease modifying therapy (29.9%). Also, 83.6% of the participants had a caregiver on a daily basis and in 96.7% of the cases the caregiver was living with the patient. The first symptom was discovered by a parent / relative / person who looked after the baby / child at home in 64.4% of the cases. In 44.4% it took more than 3 months to seek/obtain medical advice for this symptom, and in 64.4% more than 3 months to confirm the diagnosis. Furthermore, 62.7% of participants had to travel from their place of permanent residence to receive a diagnosis and 69.0% for more than 100 km. At the time of the survey, 74.6% of participants were being monitored in a neurology clinic of a university hospital and the mean age of transition to current hospital was 26.8 years (SD = 11 years). The proportion of those that experienced problems with transition to adult care was 22.4% and the most common reason mentioned (40%) was lack of center experience with their condition as well as patients (and caregivers) having to coordinate everything themselves. Of total sample, 66.0% had to wait for more than a month to start their treatment after being informed of its availability. The most common reason for delayed treatment start was its approval by the National Organisation for Health Care Services Provision (EOPYY) (68.2%). Most patients (88%) had been involved in the decision about their treatment. Of total sample, 54.0% received therapy once every 4 months, intravenously in the hospital setting. Side effects from treatment were experienced by 28% of the sample and 14.3% of them required hospitalization for their management. In total, 4.5% of the sample required hospitalization at least once during the past 12 months due to their condition.

**Table 2:** Participants’ disease characteristics, caregiver characteristics, journey from first symptom to diagnosis to current care/treatment

			*n*	%
Disease characteristics	Disease duration (years), mean (SD)		28.9 (11.3)	
	What type of SMA were you diagnosed with?	Ι	1	1.5
		ΙΙ	40	59.7
		ΙΙΙ	26	38.8
	Are you currently receiving treatment for the disease?		50	74.6
	After diagnosis and to-date, what types of treatment have you received?	Gene therapy	18	26.9
		Disease modifier	20	29.9
		Management of symptoms only	33	49.3
		Other	9	13.4
Caregiver characteristics	Since being diagnosed. has anyone supported you with your disease management (unpaid caregiver)?	No	6	9.0
		Yes, on a daily basis	56	83.6
		Yes, but not everyday	5	7.5
	Does this person, who we will call a caregiver, live with you?		59	96.7
	Does your caregiver work?		18	29.5
	Does he/she take time off work to be with you when you need him/her?		11	61.1
Journey from first symptom to diagnosis	Who discovered the first symptom?	Parent / relative / person who looked after the baby / child at home	29	64.4
		Pediatrician	7	15.6
		Other health professional (e.g. nurse, physiotherapist, etc.)	5	11.1
		Other	4	8.9
	How long did it take the family to seek/obtain medical advice for this symptom?	1–4 weeks	17	37.8
		2–3 months	8	17.8
		More than 3 months	20	44.4
	In total, how long after the first symptom did you finalize the diagnosis?	1–2 weeks	6	13.3
		1 month	8	17.8
		2 months	2	4.4
		More than 3 months	29	64.4
	Did you have to travel from your place of permanent residence to receive a diagnosis?		42	62.7
	How far?	Less than 10 km	1	2.4
		10–50 km	3	7.1
		50–100 km	6	14.3
		More than 100 km	29	69.0
		In a foreign country	3	7.1
Journey to current care/treatment	Today you are being attended at:	Neurology clinic of a general hospital	9	13.4
		Neurology clinic of a university hospital	50	74.6
		Neurology clinic of a private hospital	2	3.0
		Other	6	9.0
	Age on transition to current hospital, mean (SD)		26.8 (11.0)	
	The transition was made from the pediatric clinic that attended patient as a child		18	26.9
	Did you experience problems with transition to adult care?		15	22.4
	If yes, please state:	They didn’t admit me to the adult clinic, they found excuses to delay it	4	26.7
		I was referred to different clinics and other hospitals until I ended up here	4	26.7
		They had no experience with my condition and we had to coordinate everything	6	40.0
		Other	4	26.7
	Regarding your pharmaceutical treatment, how many days after being informed of its availability and suitability, did you start treatment?	1 week	4	8.0
		15 days	4	8.0
		1 month	8	16.0
		> 1 month	33	66.0
		Immediately. In experimental stage	1	2.0
	Did you experience problems/delays in receiving your (drug) treatment?		22	44.0
	What were they related to?	Treatment approval from public insurance	15	68.2
		Availability of treatment (e.g. delayed import from abroad)	9	40.9
		Availability of hospital / clinic (appointment) for the administration of the treatment	9	40.9
		Other	3	13.6
	Were you involved in the decision about your treatment? Have you expressed your preferences?		44	88.0
	How often do you take your medication?	Daily	23	46.0
		Once a month	0	0.0
		Once every 4 months	27	54.0
		Other	0	0.0
	Is it admninistered in the hospital (intravenous) or at home (subcutaneous / oral)?	At the hospital	27	54.0
		At home	23	46.0
		Both	0	0.0
	Did you experience any side effects from the treatment?		14	28.0
	If yes, need for hospitalization for their management		2	14.3
	In total, in the last 12 months, did you require any hospitalization for your condition (unrelated to side effects)?		3	4.5

Participants’ financial burden is presented in Table 3. Participants spent on average more than EUR 300 a month to cover costs related to their condition. In the majority of cases, these referred to services which supported their therapy, such as physiotherapy (71.6%). Patients who had to travel to get a diagnosis, covered the cost out of their pocket in an overwhelming 95.2% of the cases, whilst 55.2% had to pay out-of-pocket for any health services related to the diagnosis, with medical visits (91.9%) and diagnostic tests (73.0%) being the most common.

**Table 3:** Participants’ disease-related financial burden

	*n*	%
Average monthly financial burden due to condition		
EUR 0	6	9.0
EUR 1-100	5	7.5
EUR 100–200	16	23.9
EUR 200–300	6	9.0
EUR 300+	34	50.7
These costs relate to:		
Nursing/Health Services	22	32.8
Treatment support services	48	71.6
Medication	18	26.9
Family/home care expenses for activities that I can no longer perform	30	44.8
Home tuition expenses	1	1.5
Other	12	17.9
In order to receive a diagnosis, did you cover travel costs yourself?	40	95.2
Did you have to pay out-of-pocket for any health services related to the diagnosis?	37	55.2
For which?		
Medical visit	34	91.9
Medical visit (co-payment only)	3	8.1
Diagnostic tests	27	73.0
Diagnostic tests (co-payment only)	3	8.1
Pharmaceutical treatments	7	18.9
Pharmaceutical treatments (co-payment only)	3	8.1
Other	2	5.4

Of total sample, 70.4% of patients’ caregivers attended physiotherapy sessions, due to the need to carry additional weight. In 42.1% of the cases, this expense was covered by social insurance (Table 4). Another 52.2% of the sample were on regular food supplements, with this expense being covered by public health insurance in 1 in 5 cases (22.9%). The largest out-of-pocket expenditure referred to physiotherapy/occupational therapy with a median value of EUR 200 per month (IQR: EUR 120–240).

**Table 4:** Participants’ disease management expenses

	Use		Expense covered by insurance			Monthly out-of-pocket amount spent (€)		
	*n*	%		*n*	%		Mean (SD)	Median (IQR)
Food supplements	35	52.2		8	22.9		51.5 (22.9)	50 (30–60)
Physiotherapy/occupational therapy	37	55.2		6	16.2		217.9 (177.3)	200 (120–240)
Speech therapy	0	0		-	-		-	-
Nutritional monitoring/assessment/intervention	4	14.8		1	25		46.7 (11.5)	40 (40 ─ 60)
Orthopedic monitoring / tests (e.g. DEXA)	6	22.2		3	50		83.3 (57.7)	50 (50 ─ 150)
Dental / orthodontic follow-up / intervention (e.g. due to jaw problems)	9	33.3		4	44.4		73 (72.6)	50 (50 ─ 50)
Psychological support (for me or my partner)	9	33.3		3	33.3		121.7 (63.4)	100 (80 ─ 200)
Physiotherapy (for me or my partner – due to need to carry extra weight)	19	70.4		8	42.1		186.4 (103)	160 (100 ─ 240)

Essential aids used by participants’ and their reimbursement are presented in Table 5. Almost all participants (91.7%) owned a wheelchair and 54% had a special van/vehicle that allows the transport of a wheelchair. From those who had a wheelchair, 24.5% had paid for it on their own, while the corresponding percentage for the van was 88%.

**Table 5:** Availability and reimbursement of necessary aids

Type of aid	Have it in house	If yes, was the cost of acquiting it reimbursed by public insurance?			Out-of-pocket amount spent (€)
		No	In part	In full	
	*n*(%)	*n*(%)	*n*(%)	*n*(%)	Mean (SD)
Simple Continuous Positive Airway Pressure (CPAP) device	3 (7)	0 (0)	1 (33.3)	2 (66.7)	-
Simple Biphasic Positive Airway Pressure (BiPAP, BiPAP S/T) device	9 (20.5)	0 (0)	3 (37.5)	5 (62.5)	1,350 (1,693.4)
Volume/pressure ventilator	8 (17.8)	0 (0)	5 (62.5)	3 (37.5)	2,100 (2,515.9)
Orthotic means	10 (23.3)	1 (10)	6 (60)	3 (30)	900 (517.7)
Knee guards	3 (7.3)	2 (66.7)	0 (0)	1 (33.3)	
Shoulder guards	0 (0)	-	-	-	
Splints	13 (28.3)	3 (27.3)	7 (63.6)	1 (9.1)	1,262.5 (1,261.4)
Wheelchair	55 (91.7)	12 (24.5)	29 (59.2)	8 (16.3)	3,945.9 (3,399.6)
Special van/vehicle that allows the transport of a wheelchair	27 (54)	22 (88)	3 (12)	0 (0)	16,750 (12,089)

**Fig. 1:**
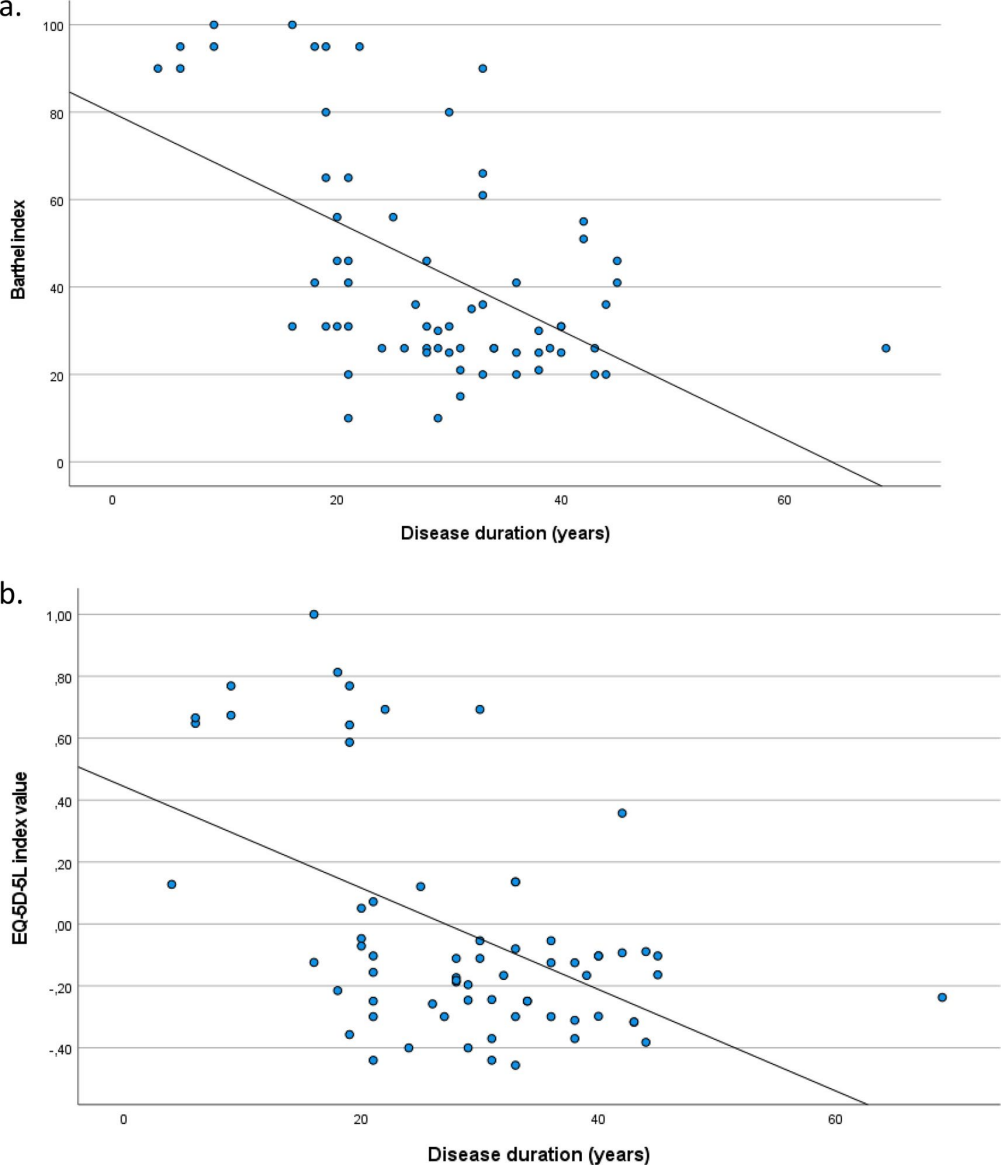
Association of disease duration with Barthel index (**a**) and EQ-5D-5 L index (**b**)

Mean value of the Barthel index was 43.9 (SD = 26), of the EQ-5D-5 L index − 0.03 (SD = 0.37) and of the VAS 66.6 (SD = 22.3). VAS scale was not significantly correlated with neither Barthel index (*r* = 0.12; *p* > 0.05) nor EQ-5D-5 L index (*r* = 0.21; *p* > 0.05), while there was a significant positive correlation between Barthel and EQ-5D-5 L indexes (*r* = 0.90; *p* < 0.001). When Barthel index was associated with participants’ characteristics, via multiple regression analysis, it was found that married patients had significantly lower Barthel index than unmarried ones (Table 6). Additionally, greater disease duration was significantly associated with lower Barthel index (Fig. 1a) and lower EQ-5D-5 L index (Fig. 1b). Similarly, greater average monthly economic burden due to the condition was significantly associated with lower EQ-5D-5 L index, while greater educational level was significantly associated with greater EQ-5D-5 L index (Table 6).

**Table 6:** Multiple linear regression analysis results (in a Stepwise method) for Barthel index and EQ-5D-5 L index value

Dependent variables	Independent variables	β+	SE++	*P*
Barthel index F(2,64) = 19.30; *p* < 0.001; R^2^ = 0.36	Disease duration (years)	-1.25	0.23	< 0.001
	Family status (married vs. unmarried)	20.65	7.14	0.005
EQ-5D-5 L index value F(3,63) = 13.32; *p* < 0.001; R^2^ = 0.36	Disease duration (years)	-0.014	0.003	< 0.001
	Educational level	0.11	0.04	0.004
	Average monthly financial burden due to disease	-0.08	0.03	0.004

^+^regression coefficients; ^++^standard error

## Discussion

This survey reports on the patient journey and experience with health care services of adult patients with SMA in Greece. Results confirm the complicated journey of patients with SMA, especially as they transition from pediatric to adult care, as well as their financial and QoL burden. More specifically, adult patients shoulder a substantial amount of out-of-pocket expenses on a monthly basis, primarily to cover medical services such as physiotherapy and occupational therapy. Furthermore, the majority of participants had to travel to obtain a diagnosis, with most of them travelling for substantial distances at own cost. In addition, a number of aids necessary for the ongoing management of their condition is paid out of their pocket, whereas food supplements are covered almost exclusively by patients themselves. This adds further strain and impacts on their QoL as evidenced by lower EQ-5D-5 L results. On the contrary, attaining a higher education appears to have a positive correlation with patient QoL, underlying the importance of SMA patient integration in social and educational life from very early on.

Our findings are in line with previous work, which reported on the grave impact of financial burden on SMA patient QoL [22] which limit the ability of the household to cover other basic needs. In addition, Peña-Longobardo et al. [23] highlighted that direct non-healthcare costs across Europe ranged between 79 and 86% of total SMA cost with informal care costs being the main component of these costs. Their findings confirm older results from Spain [24] that attributed 67.8% of total SMA costs to direct non-healthcare costs. Of these, informal caregiving constituted the major cost component. In Greece, informal caregiving, particularly by family members, may be provided on an unpaid basis, primarily due to strong familial bonds and cultural context, yet, were it to be evaluated in monetary terms, it would constitute a very large expenditure: our findings report that 83.6% of participants have a caregiver on a daily basis and in almost all (96.7%) of the cases he/she is living with the patient full time. This creates additional burden for the SMA patient and caregiver, and is potentially related to disease-related productivity losses, including absenteeism, invalidity or changes in their working situation [25].

With respect to the actual patient journey, recognizing symptoms in a timely manner to initiate the disease management process may be extremely challenging in the context of lack of universal screening. The burden falls largely on families and caregivers, who do not have the specialized knowledge to recognize symptoms of SMA or even assert a behavior as abnormal. This may result in delayed diagnosis, which inevitably impacts on the prospects of successful early intervention. In our survey, the majority of patients reported the first symptom being discovered by a parent / child carer at home, and then a period of at least 6 months to seek medical advice and confirm the diagnosis. Our findings are in line with those previously reported by Pera et al. [2], which confirm that over 63% of cases were being recognized by parents first, with a mean time between symptom diagnosis and genetic testing of between 1.94 and 16.8 months, depending on SMA type.

Three quarters of participants in our survey reported being monitored in a university neurology clinic, after spending, on average, at least a decade transitioning from pediatric care. The reason both for the delay in transition and the current situation of being monitored by a university clinic is mainly the lack of knowledge or experience of the rest of the healthcare system with such a rare condition. This, together with lack of central coordination and guidance towards specialized centers, leads patients and their caregivers to having to search for and coordinate their care themselves. This disparity in care organization and delivery between child and adult health services may lead to unmet health care needs, with SMA adult patients finding themselves in a space between pediatric and adult health care worlds [26]. In addition, and in light of newly developed and available disease modifying treatments that are prolonging survival, there is a dire need for strategies to help people with SMA navigate the transition from adolescent to adult life [13], improving their care and empowering this underserved population [27] with solutions that can help the health care system become more age-appropriate, more comprehensive, integrated, and robust. A recent survey from Australia [28] highlighted precisely those difficulties faced with adult health care services, as almost all participants reported hardship with identifying and accessing specialists and multidisciplinary clinics for adults with SMA, or perceived available services as inadequate. This, it was reported, carried the risk of disengagement from healthcare services, until significant declines in muscle strength and functional ability or the opportunity to access a new treatment motivated frustrated adult patients to re-engage with the system.

Spatial distribution of such services is also critical. A recent survey from Romania confirmed that smaller distance from point of care positively impacts outcomes in SMA patients [29]. Therefore, it is important to ensure care integration and then, managed decentralization, for specific services, as needed, to meet patient needs for access to specialized adult care.

Further highlighting this need, it appears that, in specialized centers, patient voice is heard. The vast majority of our survey participants treated in specialized centers reported being informed of options and participating in the treatment decision. This is especially critical both for patient adherence to treatment and for meeting patient psychological needs and should be encouraged across the patient pathway. Autonomy supportive healthcare professionals that take on a patient-centered approach and promote partnership and collaboration with their patients by active listening and seeking patient input, whilst also respecting patients’ preference and choice, are shown to actively contribute to improved patient outcomes [30].

The majority of our sample had to wait for more than a month to start a treatment suitable for their status, due to, primarily bureaucratic delays related to public insurance’s approval of its reimbursement. Given the impact to patient outcomes of early initiation of treatment [31] consideration should be given to optimization of approval processes and the removal of any bureaucratic steps to expedite access to appropriate treatment for all SMA patients, including adults.

Finally, in our survey, higher education appears to be positively correlated with improved QoL. 40% of our sample had completed university education, which is close to the 33–66% reported in the review by Wan et al. [32]. While SMA can significantly impact physical functioning, individuals with higher education often report better mental well-being and adaptation to disease-related challenges. In a study by Wohnrade et al. [33] ≥ 12 educational years were positively correlated with significantly higher values in the SF36 Vitality dimension. Higher levels of education are seemingly also correlated with improved employment opportunities, with over 65% of our sample being employed, a number higher than those reported in the literature (18 to 49%, Wan et al. [32]). Employment, as a factor contributing to financial independence and overall autonomy is considered a critical priority by adults with SMA [34], is an important aspect of inclusive policies, and should be actively encouraged by the State, to overcome persistent barriers to work and education reported elsewhere in the literature [35].

This survey is aimed at reporting on patient journeys of adult patients with SMA and as such addresses a gap in literature, which is, understandably, more extensive on caregivers’ experience with pediatric SMA. Though the challenges of finding out the presence of SMA and navigating disease management very early on in a child’s life are profound and merit scientific and research focus, there is a need to showcase the experience of SMA adult patients and the impact of the condition on their financial outlook and QoL. We hope this paper contributes to this aim and sheds light on the importance of continuity in care provision especially in rare genetic conditions.

## Limitations

All data used in this survey are self-reported. This includes diagnosis as SMA patient and type of SMA. All patients were recruited by invitation from MDA Hellas amongst their members, thus minimizing the risk of any misrepresentation of disease status. As this was a convenience sample, we realize it may not be representative of total adult SMA population in Greece. Patients were asked to report on their SMA type, which may be less representative of patients’ function in adulthood. Nonetheless, this enabled comparisons with findings in the literature as well as results of a parallel survey performed in underage patients, through their caregivers, in Greece. Some of the data refer to events over a decade ago and may, thus, be affected by recall bias. We would like to recognize that individual journeys in rare diseases may be extremely varied, and each patient has a unique experience with the health care system. This survey aims to combine these experiences across the continuum of care to report on key challenges and barriers, in order to inform and facilitate an evidence-based advocacy for their resolution. As a result, individual circumstances may divert from the generally described patient journey.

## Conclusion

SMA is a rare genetic disorder that impacts gravely on patient and family QoL. Our survey showcases that as the disease management paradigm evolves with the wider availability of disease modifying treatments and patients live well into adulthood, healthcare systems need to reconsider their pathways to care to ensure such updates are integrated and patients can optimize use of available resources in the most efficient manner.

## Data Availability

The authors are committed to sharing the full survey with qualified external researchers. The requests are to be made to the corresponding author and will be appraised based on scientific merit.

## Abbreviations

EOPYY: National Organisation for Health Care Services Provision
IQR: Interquartile range
SD: Standard Deviation
SE: Standard errors
SMA: Spinal muscular atrophy
SMN1: Survival of motor neuron 1 gene
VAS: Visual Analogue Scale
